# Single‐room maternity care: Systematic review and narrative synthesis

**DOI:** 10.1002/nop2.586

**Published:** 2020-08-31

**Authors:** Elena Ali, Jill M. Norris, Marc Hall, Deborah E. White

**Affiliations:** ^1^ Faculty of Nursing University of Calgary Calgary AB Canada; ^2^ University of Calgary in Qatar Doha Qatar

**Keywords:** delivery rooms, labor delivery recovery postpartum, literature review, maternal health services, nursing, rooming in, single room maternity care, systematic review

## Abstract

**Aim:**

To describe the single‐room maternity care model and evaluate its influence on patient, provider and system outcomes.

**Design:**

Mixed‐method systematic review and narrative synthesis.

**Methods:**

We conducted searches of MEDLINE, CINAHL, Web of Science, Cochrane Database of Systematic Reviews, and the grey literature from January 1985–August 2018, yielding 151 records. Pairs of reviewers independently applied the inclusion criteria using a standardized screening tool to both titles/abstracts and full texts. Overall, 13 studies were retained.

**Results:**

Most studies of single‐room care were from the United States and Canada, and assessed costs, patient satisfaction and/or provider satisfaction. Studies used cross‐sectional and/or pre–post comparative, retrospective descriptive and qualitative designs. Methodological quality of quantitative studies was generally weak, and few studies conducted inferential statistics. Maternal satisfaction with the single‐room maternity model was positive across the studies; however, healthcare provider satisfaction was mixed.

## 1 INTRODUCTION

Single‐room maternity care (SRMC)—also known as labour‐delivery‐recovery‐postpartum (LDRP) care—was developed as an alternative to the traditional labour and delivery suites during the family‐centred care movement, which promoted birth as a normal family process (Stolte, Myers, & Owen, 1994). First introduced in South Africa in the 1970s (Notelovitz, 1978), SRMC has been increasingly taken up by hospitals in North America, Europe and Australia (Phillips & Fenwick, 2000; Zwelling & Phillips, 2001). For example, 23% of maternity rooms in Canadian hospitals were assigned as LDRP in 2012, compared with 7% in 1993 (Public Health Agency of Canada, 2012). In the SRM unit, a childbearing woman and her family stay in one room from admission until discharge from the hospital, without transferring between units, and the newborn remains with the family at all times to enhance family beginnings (Harris, Farren, Janssen, Klein, & Lee, 2004; Phillips & Fenwick, 2000). SRM units provide a home‐or bedroom‐like environment, with medical equipment stored out of sight. This contrasts with traditional maternity care where labouring women are first admitted to the labour and delivery unit, and then transferred to the postpartum unit within hours of birth. Traditional maternity rooms are designed like other hospital rooms—small, with a central bed and medical equipment in view.

## 2 BACKGROUND

While the interest in SRMC has grown, the accompanying evidence base to support it is unclear. In their seminal description of SRMC care published by the Association of Women's Health, Obstetric and Neonatal Nurses, Phillips and Fenwick (2000) provided several anecdotal case studies of the positive impact of the model; however, these findings arose from their private consulting work. Hospital single rooms in general may have a moderate positive effect on patient‐reported outcomes (e.g. satisfaction, noise level, quality of sleep and privacy), but the impact on patient safety and health outcomes was equivocal (van de Glind, de Roode, & Goossensen, 2007). Results of a Cochrane review of nine randomized trials of alternative birth settings for labour and delivery (Hodnett, Downe, & Walsh, 2012)—such as birth centres, ambient rooms and Snoezelen rooms—suggested that there were lower rates of medical interventions and greater maternal satisfaction with these settings. Similarly, a review of single‐family rooms in neonatal intensive care units indicated that families preferred single rooms for the increased privacy and reduced noise, and patients exhibited shorter length of stay and fewer infection (Shahheidari & Homer, 2012). While healthcare providers valued single rooms, they reported concerns about reduced patient visibility, staff communication, workload and physical demands of their job with further distance between rooms. More recent concerns highlight the potential impact of a sensory‐deprived environment on preterm infants’ development and feelings of isolation by parents as well as staff (Dunn, MacMillan‐York, & Robson, 2016).

The decisions around the design of maternity care units are complex and trade‐offs may be necessary between design considerations, staff and patient preferences, and economic demands. Understanding the impact SRMC may have on patients, care providers and overall healthcare system may provide directions for policymakers’ decisions about implementation of this model. The objective of this review was to describe SRMC and evaluate its influence on patient, provider and system outcomes.

## 3 THE STUDY

### 3.1 Design

We conducted a mixed systematic review (Centre for Reviews & Dissemination, 2009) and narrative synthesis (Popay et al., 2006) given the known heterogeneity of the single‐room maternity literature. The PRISMA statement (Moher et al., 2009) was used to guide the reporting. Our multidisciplinary review team had expertise in healthcare organizations and workforce, maternity care and knowledge synthesis.

### 3.2 Methods

We searched MEDLINE (Ovid), CINAHL, Web of Science, and the Cochrane Database of Systematic Reviews. Searches were adapted for each database, including appropriate subject headings: single room, single family, labor delivery recovery postpartum, labor delivery recovery, rooming in, delivery room, delivery, obstetric, maternal health services, maternal care, health services (Table 1 for MEDLINE search strategy). Searches were limited to English‐language publications from 1985–August 2018. Grey literature searches were conducted through electronic databases (Web of Science Conference Proceedings Citation Index—Science and Social Science & Humanities—1990‐present; ProQuest Dissertation and Theses; Google; Google Scholar), hand searches of relevant journals (e.g. Journal of Nurse Midwifery, Journal of Clinical Nursing, Birth), professional organization websites (e.g. Association of Women's Health, Obstetric and Neonatal Nurses; Society of Obstetricians and Gynaecologists of Canada; Public Health Agency of Canada) and reference lists of included studies. Search results were imported into EndNote for duplicate removal.

**Table 1:** Medline search strategy

Search strategy	
1	exp Rooming In/
2	exp Delivery Rooms/
3	exp Delivery, Obstetric/
4	exp Maternal Health Services/
5	1 or 2 or 3 or 4
6	single room.mp
7	single‐room.mp
8	single family.mp
9	single‐family.mp
10	labor delivery recovery postpartum.mp
11	labor‐delivery‐recovery‐postpartum.mp
12	labor‐delivery‐recovery.mp
13	labor delivery recovery.mp
14	6 or 7 or 8 or 9 or 10 or 11 or 12 or 13
15	5 and 14

We included studies that met the following criteria: (a) participants included mothers, infants, families or providers; (b) described or evaluated SRMC; (c) assessed patient‐, provider‐ or system‐level outcomes or perceptions of mothers, families and providers; (d) English‐language; and (e) peer‐reviewed primary study of any design. Studies were excluded if the primary focus was not on the impact of single‐room maternity (e.g. simulation or breastfeeding conducted in single‐room maternity), single rooms from other settings (e.g. neonatal intensive care units, other areas of the hospital) or care models that only included labour, delivery, recovery and not postpartum.

Before the screening process, we pilot tested the screening tool in Microsoft Excel with the review team. Subsequently, two reviewers independently applied the inclusion criteria using the standardized screening tool to both abstracts (98.3% agreement) and full texts (87.5% agreement). Disagreements were resolved by consensus.

Studies were not excluded based on quality. For quantitative studies, we used the Effective Public Health Practice Project Quality Assessment Tool (EPHPP; Thomas, Ciliska, Dobbins, & Micucci, 2004); six domains were assessed as *strong, moderate, weak* or *not applicable*. For qualitative studies, we used the Critical Appraisal Skills Programme Qualitative Checklist (CSAP, 2014), and the 10 domains were assessed as *no, yes* or *can't tell*. For mixed methods studies, we used both the quantitative and qualitative quality appraisal tools. Two reviewers divided the studies and independently appraised studies for methodological quality. The accuracy of the quality assessment was verified by a second independent reviewer for each study, and disagreements were resolved by a third reviewer.

### 3.3 Analysis

The review team pilot tested the data extraction form in Microsoft Excel prior to extracting data. Two reviewers divided the studies and independently extracted data items including the following: study year, country, funding sources, study design, participants (eligibility, response rates and characteristics), description of SRMC and comparison units (site, environment, care model, training), data collection and analysis, and findings. A second reviewer verified all extracted data to ensure accuracy, and any disagreements were resolved through discussion between reviewers. Given the heterogeneity of studies, statistical pooling of the quantitative data for meta‐analysis was not possible. We used a narrative approach (Popay et al., 2006) to synthesizing the included studies and calculated the frequency of study characteristics for presentation in a tabular format.

### 3.4 Ethics

This review was exempt from ethical approval.

## 4 RESULTS

### 4.1 Study characteristics

**FIGURE 1:**
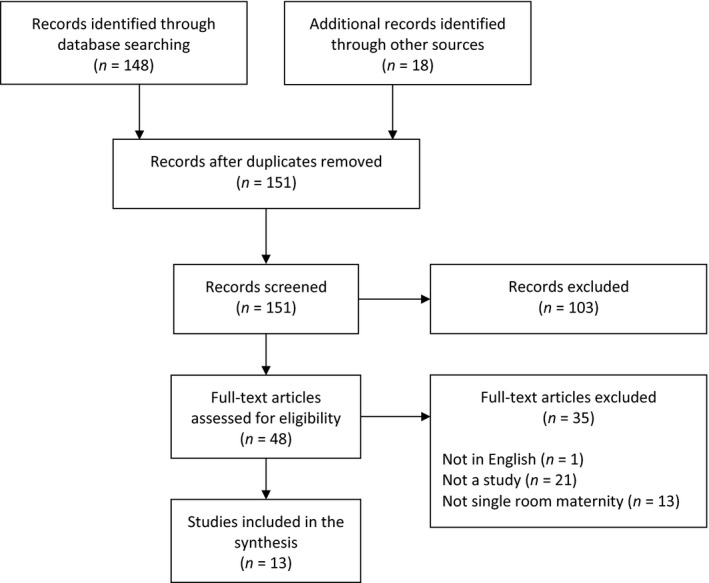
Study flow diagram

From 166 records, we screened 151 abstracts for inclusion (Figure 1). A total of 48 full‐text articles were reviewed, and 13 studies were retained for the final synthesis (see Table 2 for study characteristics). There were no controlled studies; studies used predominantly before–after and cross‐sectional comparative (*N = *6) or descriptive designs (*N = *5). Most of the included studies were published prior to 2004 (*N = *10) and were from North America (*N = *11). Across the studies, 195 healthcare providers and 1,315 patients were surveyed or interviewed, and 15,404 patient records were reviewed (see Table 3).

**Table 2:** Characteristics of included studies

Study characteristic	*N*	%
Country		
United States	6	46.2
Canada	5	38.5
Australia	1	7.7
Netherlands	1	7.7
Year		
1987–1999	6	46.2
2000–2004	4	30.8
2005–2009	1	7.7
2010–2015	2	15.4
Design		
Before‐after, cross‐sectional comparative	2	15.4
Before‐after	2	15.4
Cross‐sectional comparative	2	15.4
Descriptive	5	38.5
Mixed methods	1	7.7
Qualitative	1	7.7
Sample		
Patients	9	69.2
Providers	6	46.2
Nurses	4	30.8
Physicians	1	7.7
Various	1	7.7
Data sources		
Survey	8	61.5
Administrative databases, patient records	6	46.2
Interviews	2	15.4
Outcomes		
Patient satisfaction, perceptions	5	38.5
Provider satisfaction, perceptions	6	46.2
Clinical outcomes	4	30.8
Costs	4	30.8

**Table 3:** Included studies

First author	Year	Design	Participants [Group]	Data sources (Response rate)	Setting	Description of single‐room maternity unit
Janssen	2000	Before‐after, cross‐sectional comparative	221 patients [historical TM] 205 patients [SRM] 104 patients [concurrent TM]	Survey	Vancouver, BC BC Women's Hospital	7 single rooms: natural light, bed for labour support person, large bathtub with shower, soundproofing, family lounge space for preparing and eating meals Staffing: Nurse‐patient ratio is 1:1 in labour and 1:4 in PP period; communicate via in‐house wireless telephone system; competency‐based perinatal nursing training programme Other: 8‐room low risk delivery suite, with postpartum rooms
Janssen	2001	Cross‐sectional comparative	20 nurses [SRM] 26 nurses [TM] 26 nurses [PP]	Survey (100%)		
Harris	2004	Before‐after, cross‐sectional comparative	583 patients [SRM] 393 patients [concurrent TM]	Administrative and perinatal database		
			34 physicians [SRM before‐after, 1 year]	Survey (42.5%)		
Janssen	2005	Before‐after	19 nurses [SRM training before‐after]	Survey		
Janssen	2006	Cross‐sectional comparative	150 patients [SRM] 281 patients [TM]	Survey		
Olson	2004	Descriptive (cross‐sectional)	343 patients [SRM]	Survey (43.5%)	Rochester, MN Birth Centre in Olmsted Community Hospital	17 single rooms: foetal monitors, oxygen, suction, newborn warmers, and cribs, removable equipment Staffing: Orientation programme developed to cross‐train nurses
			28 nurses [SRM]	Survey (68.2%)		
Hickey	1998	Descriptive (survey development)	30 nurses [SRM]	Survey	New Haven, CT Community hospital	Single rooms Staffing: Nurses were cross educated Other: traditional delivery rooms and caesarean section rooms
Williams	1989	Descriptive (retrospective)	7,447 patients [SRM]	Patient records	Tampa, FL Teaching hospital	12 single rooms: pleasant home‐like décor, labour beds that can be converted to procedure bed, electronic foetal monitors, oxygen and vacuum suction outlet, infant warmer Other: 4 operative delivery room, 6‐bed postsurgical recovery room, 6‐room perinatal special care unit with invasive maternal cardiac monitoring capabilities
Permezel	1987	Descriptive (retrospective)	1,794 patients [SRM]	Patient records	Melbourne, AU Royal Women's Hospital Family Birthing Unit	5 single rooms: wall to wall carpeting, wall paintings, double bed, medical equipment is kept out of sight Other: traditional labour ward and caesarean section rooms
Gerrits	2013	Descriptive (retrospective)	1,522 patients [TM, 2005] 1,790 patients [SRM, 2008] 1,875 patients [SRM, 2009]	Electronic patient database	Nijmegen, NL Canisius‐Wilhelmina Hospital	13 single rooms: non‐medical atmosphere, medical equipment is kept out of sight
Drum	1992	Before‐after	NR patients [SRM before, after]	Survey	Salisbury, MD 300‐bed acute care facility	NR
				Administrative database		
			12 staff [SRM before, after]	Survey		
Bergeron	1988	Mixed methods comparative	NR	Administrative database	Kansas, Missouri, and Louisiana, US 6 healthcare facilities	NR
				Interviews		
Rogner	2011	Qualitative	11 patients [SRM]	Interviews	NR	NR

Abbreviations: NR, not reported; PP, postpartum; SRM, single‐room maternity; TM, traditional maternity.

### 4.2 Quality assessment

The methodological quality of quantitative studies was generally weak (Table 4); one qualitative study had strong methodological quality (Rogner, 1995) (Table 5). One study controlled for potential confounders; Harris et al. (2004) reported group differences by ethnicity, nulliparous, gestational age at admission, previous caesarean section and delivery caregiver (*p <* .05). Four studies used surveys with evidence for internal reliability (Hickey, 1994; Janssen, Dennis, & Reime, 2006; Janssen, Harris, Soolsma, Klein, & Seymour, 2001; Janssen et al., 2005). Harris et al. (2004) used the same survey as an earlier study (Janssen et al., 2001), but with a different population (physicians) and the reliability of the tool was not reported. Beyond describing pilot testing survey tools for face validity (Bergeron, 2001; Janssen et al., 2001; Janssen, Klein, Harris, Soolsma, & Seymour, 2000), two studies provided evidence for construct validity through resulting factors from exploratory factor analysis (Janssen et al., 2005, 2006).

**Table 4:** Quality appraisal of quantitative studies

Study	Selection bias	Study design	Confounders	Blinding	Data collection methods	Withdrawals, drop outs
Janssen et al. (2000)	1	1	2	3	3	2
Janssen et al. (2001)	2	1	2	1	1	1
Harris et al. (2004)	2	1	2	3	1	3
Janssen et al. (2005)	2	1	1	2	1	1
Janssen et al. (2006)	1	1	2	3	1	2
Olson and Smith (1992)	3	3	1	3	3	4
Hickey (1994)	1	3	3	3	1	4
Williams & Mervis, 1990	2	2	3	3	2	N/A
Drum (2011)	3	3	1	3	3	3
Gerrits et al. (2013)	1	3	3	3	3	4
Bergeron (2001)^a^	3	3	N/A	3	3	N/A
Permezel et al. (1987)	1	3	3	3	3	4

Note

1, strong; 2, moderate; 3, weak.

Abbreviation: N/A, not applicable.

^a^ Mixed method study.

**Table 5:** Quality appraisal of qualitative studies

Study	Aims	Methodology	Design	Recruitment	Data collection	Researcher‐participant relationship	Ethical issues	Data analysis	Findings	Value
Rogner (1995)	1	1	1	1	1	1	1	1	1	1
Bergeron (2001)^a^	1	1	1	2	2	2	2	2	1	2

Note

0, no; 1, yes; 2, can't tell.

^a^ Mixed method study.

### 4.3 Description of single‐room maternity care

Overall, descriptions of the environment, nursing preparation and staffing requirements were sparse (Table 3). Six SRMC units were described across 10 studies. Units housed between 5–18 single patient rooms.

#### 4.3.1 Environment

Five studies briefly highlighted the physical environment of the units (Gerrits, Hosson, Semmekrot, & Sporken, 2013; Janssen et al., 2000; Olson & Smith, 1992; Permezel, Pepperell, & Kloss, 1987; Williams & Mervis, 1990). The atmosphere in the patient room was described as “non‐medical, restful, private and safe” (Gerrits et al., 2013), and “home‐like” (Permezel et al., 1987; Williams & Mervis, 1990), with a “pleasant” décor (Williams & Mervis, 1990). Comfortable maple furnishings with modern fabrics, wall art (Janssen et al., 2000), wall to wall carpeting (Permezel et al., 1987) and large windows providing natural daylight (Janssen et al., 2001) were some of the décor features. The rooms were “large enough” for family members and the healthcare staff to move around comfortably (Janssen et al., 2001). Common amenities included television, radio, telephone and stereos. All rooms had a private bathroom with a walk‐in shower. Some bathrooms had bathtubs to accommodate for hydrotherapy during labour and water births (Janssen et al., 2000). The rooms were equipped with special obstetrical beds that convert to various delivery and recovery positions, a bassinet and/or an infant warmer. The rooms contained foetal heart rate monitors, and oxygen, suction, and medical gas outlets. A comfortable chair or a sofa was available for the family members and support persons in the curtained‐off area of the room. The labour and delivery equipment was brought into the patient's room as needed and removed after the delivery (Janssen et al., 2000). Smaller medical equipment (e.g. intravenous solutions, gloves, suturing supplies) were stored in steel carts to avoid a “surgical atmosphere” in the room (Gerrits et al., 2013). To keep the noise levels low, some units had soundproofing installed in the walls (Janssen et al., 2001). Some units had a family lounge, where family members can prepare meals or watch television (Janssen et al., 2000). Operating rooms were normally not located on the same hospital floor with the single‐room maternity unit.

#### 4.3.2 Nursing training

Three studies commented on specific nursing training for SRMC (Hickey, 1994; Janssen et al., 2005; Olson & Smith, 1992). The care model requires nursing staff to be cross‐trained to ensure competency in labour, delivery, postpartum and infant care skills (Janssen et al., 2001). Janssen et al. (2005) evaluated a competency‐based perinatal education programme that prepared nurses to practice in the single‐room maternity unit. The programme included a choice between classroom lessons or self‐learning modules tailored to the individual learning needs in the areas of foetal and neonatal health assessments, labour/delivery skills, postpartum skills and cardio‐pulmonary resuscitation. On completion of the learning activities, a nurse practiced newly acquired clinical skills with a preceptor. Nurses were given a 2‐day orientation to cover topics like emergency transfer to the operating room and working with support staff. Additionally, the nursing instructor was available daily for the new staff. A “cross‐sectional” programme for the SRMC nurses that included a week‐long classroom‐based instruction led by a nurse educator was described (Hickey, 1994). The programme did not have a standard curriculum. To be considered cross‐trained in labour/delivery, neonatal and postpartum care, nurses had to successfully complete an examination. Olson and Smith (1992) mentioned that nurses were cross‐trained through an orientation programme, but no specifics were provided about the programme.

#### 4.3.3 Nurse self‐efficacy and competence

Janssen et al. (2005) evaluated nurse self‐efficacy and competence before and 6 months after the nursing perinatal competency training programme. Mean total scores improved for both self‐efficacy (mean = 105.4 vs. mean = 110.9, *p =* .007) and competency (mean = 96.5 vs. mean = 102.2, *p =* .017).

#### 4.3.4 Staffing

Similar to a traditional care unit, SRMC nurse to patient ratio was 1:1 for women in active labour, and 1:4 for mother and infant dyad in the postpartum period in a seven‐bed SRMC unit (Janssen et al., 2001). The unit was staffed with three nurses, supported by a patient services clerk and patient services aid. Nurses and support staff communicated by means of wireless telephone system.

### 4.4 Patient satisfaction

Patient satisfaction with single‐room maternity was evaluated in three studies (Janssen et al., 2000, 2006; Olson & Smith, 1992). Janssen et al. (2000) surveyed three groups of women who were eligible for SRMC: (a) women admitted to traditional care for the 3 months prior the SRMC unit opening (i.e. historical); (b) women admitted to traditional care after the SRMC unit opened (i.e. concurrent); and (c) women admitted to the SRMC unit for the first 6 months after opening who preferred traditional care or had no preference. The survey collected data about adequacy of information and support (i.e. being with friends and family), privacy needs, physical environment, nursing care, teaching, infant feeding and discharge planning. Mean satisfaction scores in SRMC patients were significantly higher in each of the concepts compared with current traditional care patients (*p <* .05), except for common hospital issues such as noise level, hospital food and inconsistency of discharge information.

In a follow‐up study, Janssen et al. (2006) developed the 40‐item Care in Obstetrics: Measure for Testing Satisfaction (COMFORTS) scale. The six subscales resulting from exploratory factor analyses included the following: confidence in newborn care, postpartum nursing care, provision of choice, physical environment, respect for privacy, and labour and delivery nursing care. Mean scores were significantly higher in the SRMC group (mean = 181.05, *SD* 15.26) than the traditional care group (mean = 164.25, *SD* 19.39) across the total score (maximum possible score of 200) and in each subscale (*p <* .05). Multiparous women across both groups rated confidence in newborn care higher than primiparous women (mean = 42.8, *SD* 5.90 vs. mean = 39.7, *SD* 6.28; *p <* .001).

Olson and Smith (1992) presented results from cross‐sectional surveys that evaluated patient and staff satisfaction following the development of a 17‐room SRMC unit. Likert‐type survey items ranged from 1 (not satisfied)–5 (most satisfied). Mothers delivering in the first year of the SRMC unit were satisfied with the unit (*mean = *4.5).

### 4.5 Provider satisfaction and perceptions

#### 4.5.1 Nurse satisfaction

Olson and Smith (1992) concluded that nurses were generally satisfied with providing care in SRMC (mean = 4.0). In another study (Janssen et al., 2001), a cross‐sectional survey was administered to the SRMC nurses, labour and delivery unit nurses, and postpartum care nurses before and after the new SRMC unit was opened. The survey measured satisfaction with physical settings, quality of care and quality of nursing practice environment. Except for lighting, accessible delivery supplies, and newborn resuscitation equipment, single‐room maternity nurses reported more satisfaction with their physical space and quality of care than either one or both comparison groups (*p <* .05). Satisfaction with the nursing practice environment was not different across groups. Nurses’ responses to the open‐ended questions in the survey indicated that SRMC nurses were pleased with providing family‐centred and continuous care, pleasant physical environment, teamwork and autonomous practice.

#### 4.5.2 Physician satisfaction

Harris et al. (2004) surveyed physicians about their satisfaction with the new SRMC unit. Physicians preferred single room to traditional care unit because of less noise (*p <* .001), privacy (*p =* .01), spaciousness (*p =* .02), the ability to accommodate water therapy (*p <* .001) and family‐centred care (*p =* .02). Physicians did not significantly differ in their ratings of lighting and accessibility of delivery supplies between SRMC and traditional care unit. According to a 1‐year follow‐up survey, 78.7% (*p* = .003) of the physicians preferred to work in the single‐room maternity unit.

### 4.6 Clinical outcomes

Three studies assessed the impact of single‐room maternity on clinical outcomes of mothers and infants. Harris et al (2004) explored clinical outcomes before and after the opening of the new SRMC unit. Cross‐sectional data from the hospital perinatal databases for women who were eligible for SRMC were compared between those who were admitted to single‐room maternity and those admitted to traditional care. With the exception of lower rates of electronic foetal monitoring (45.8% vs. 52.7%, *p =* .004) and intravenous therapy (45.8% vs. 52.9%, *p =* .03) in the single‐room group, rates of intrapartum interventions and adverse outcomes were not significantly different between groups. Length of stay was shorter in single room (55.1 vs. 61.0 hr, *p <* .001). Nulliparous women in the single‐room group had significantly longer first stages of labour (12.0 vs. 10.8 hr, *p =* .008) and second stages of labour (120 vs. 90 min, *p =* .002). Neonatal outcomes did not differ between groups, with the exception of fewer 1‐min Apgar scores < 7 in single room compared with traditional care (10.3% vs. 15.8%, *p <* .001). The two other studies reported a decrease in the number of hypoglycaemias (Gerrits et al., 2013) and no change in perinatal mortality rate after SRMC was implemented (Williams & Mervis, 1990).

### 4.7 System outcomes

Four studies reported cost savings, without using inferential statistics to test for differences over time (Bergeron, 2001; Drum, 2011; Harris et al., 2004; Williams & Mervis, 1990). Harris et al. (2004) compared staffing costs from hospital administrative data from 2 years prior to opening the single room to the 2 years following the opening of the single room. The maternity programme FTE positions decreased from 206–193.7 (6% reduction), for an annual savings of $670,240. The costs for single‐room nurse training programme was $19,800 and direct costs for patients of similar acuity classified by resource intensity weightings reduced by 24% ($2,377–$1,809). The positive responses surrounding the opening of the single‐room unit resulted in the closure of a postpartum unit and three delivery rooms in favour of offering more single room. In the remaining three studies, cost data were not analysed, rather described. Drum (2011) reported a 12% decrease in labour hours, which equalled to over $533,000 in saving. Olson and Smith (1992) detailed the number of births, total expenses, total cost per bed day and total cost per disposition for six hospitals: two traditional model, two LDR and two single room. While authors concluded that single room and LDR were more cost effective than traditional model, this statement was not tested and one LDR hospital exhibited the highest total cost per bed day ($2,248) and total cost per disposition ($973). One study also described that offering single room increased private deliveries from 7%–14% (Williams & Mervis, 1990).

## 5 DISCUSSION

This is the first systematic review to appraise and synthesize the evidence for SRMC. While results suggest that mothers were satisfied with the SRMC model, most studies were methodologically weak and lacked reliable and valid tools to gather and analyse data. Moreover, there was limited exploration of costs, staffing, and provider and patient outcomes. Overall, there is limited evidence to suggest differences between the SRMC and traditional maternity care for patient and provider satisfaction, clinical or system outcomes.

When compared with traditional care, women were more satisfied with the SRMC due to the physical setting, continuity and quality of care, respect for privacy and assistance with infant feeding. However, no studies used specified effect sizes, and some only included descriptive comparisons rather than inferential statistics. Furthermore, three studies (Janssen et al., 2001, 2005, 2006) that used statistical testing and detailed instrument psychometrics were conducted exclusively at the same hospital in Western Canada. A systematic review of 25 studies (van de Glind et al., 2007) reported an overall higher patient satisfaction with noise level, quality of sleep, privacy and dignity on the various single‐room patent units (excluding maternity and psychiatric settings). While evidence suggests patient satisfaction may be increased in single‐room environments, more evaluation is needed to evaluate this specifically in a single‐room maternity environment in comparison to a traditional model of care.

Provider satisfaction was measured using self‐report surveys in small samples of nurses and physicians with variable response rates. While physicians preferred the SRMC to the traditional care, they disliked that emergency equipment and supply carts were less accessible in single‐room maternity and expressed concerns about the distance of the unit from the operating room (Harris et al., 2004). The findings about nurses’ satisfaction with the SRMC model are equivocal, some noting increase in satisfaction, while others do not. In summary, in all but one study (Stolte et al., 1994) nurses were asked to complete surveys in their work setting. As such, it is possible that to avoid being reprimanded by their employers and management, nurses’ responses may have been subjected to social desirability.

The environment in healthcare settings can have effects on clinical and psychological outcomes of patients. In Dijkstra, Pieterse, and Pruyn (2006) review, they reported positive clinical effects in patients from increased exposure to natural daylight (reduced length of stay, mortality rates, perceived stress and pain). Tanja‐Dijkstra and Pieterse (2011) noted that renovations to a psychiatric ward (i.e. lowered ceilings, light‐coloured floor tiles, warm wall colours) resulted in improved mood of the healthcare providers working on the unit. Yet, maternal and neonatal outcomes (Harris et al., 2004; Permezel et al., 1987) were not significantly different between the SRMC and traditional care. For example, Shahheidari and Homer (2012) indicated that single‐family neonatal intensive care unit (NICU) rooms were associated with reductions in length of stay, increased privacy and fewer patient infections, compared with the open‐bay designs. Similarly, Lester et al. (2014) found that infants in the single NICU rooms compared with infants in the open‐bay rooms weighed more at discharge, had greater rates of weight gain and required fewer medical procedures, less sepsis, better attention, less physiologic stress, less hypertonicity, less lethargy and less pain. While research (Bergeron, 2001; Drum, 2011; Harris et al., 2004; Williams & Mervis, 1990) indicated increased revenues attributed to space reduction, lower number of full‐time positions, decreases in labour hours and increase in number of deliveries, only one study (Harris et al., 2004) conducted statistical testing for significance in the differences. System outcomes research compared direct costs of care in open‐bay to single rooms in the NICU and shown that care can be provided in the single‐room NICU at no additional cost compared with the open‐bay room (Stevens et al., 2014).

### 5.1 Limitations

While we used a robust strategy for searching the literature, some relevant studies may have been missed. Four of the 13 studies were conducted at the same Canadian hospital, limiting the generalizability of the already small sample of evidence. Although we noted a potential positive effect of single room on patient satisfaction, it may be biased due to a portion of the studies being conducted on the same unit. Thus, we suggest that implementation of the SRMC based on practice, clinical and system outcomes may be premature.

### 5.2 Conclusion

We need to further understand the mechanisms by which SRMC may or may not lead to positive outcomes for mothers, infants and the healthcare system. Despite representing the best available evidence, the limitations inherent in the studies identify the need to conduct rigorous, high‐quality comparative studies between SRMC and traditional care. A greater understanding about SRMC model will provide data to inform those who wish to develop similar units and those who wish to use it.

A comprehensive analysis of cost and staffing data are needed to assess whether there are monetary advantages to the SRMC relative to traditional maternity care. Of the included studies, no outcomes were reported for data points beyond 1‐year postimplementation of SRMC. While the single‐room design in other areas may have an impact on clinical outcomes, it is unclear whether this can produce same benefit in the maternity care unit. Further evaluation of long‐term effects of the SRMC is required.

## CONFLICT OF INTEREST

The authors declare that there is no conflict of interest.

## AUTHOR CONTRIBUTIONS

All authors: Study design and screening; EA and MH: Data extraction and quality assessment; JMN: Data checking and quality assessment. All authors: Manuscript drafting and approval.

## ETHICAL APPROVAL

Not applicable.
